# Antioxidant-based neuroprotective effect of dimethylsulfoxide against induced traumatic brain injury in a rats model

**DOI:** 10.3389/fphar.2022.998179

**Published:** 2022-10-06

**Authors:** Ibrahim Bulama, Suleiman Nasiru, Abubakar Bello, Abdullahi Yahaya Abbas, Jinjiri Ismail Nasiru, Yusuf Saidu, Musa Samaila Chiroma, Mohamad Aris Mohd Moklas, Che Norma Mat Taib, Ali Waziri, Bilbis Lawal Suleman

**Affiliations:** ^1^ Department of Veterinary Physiology and Biochemistry, Faculty of Veterinary Medicine, University of Maiduguri, Maiduguri, Nigeria; ^2^ Department of Human Anatomy, Faculty of Medicine and Health Sciences, Universiti Putra Malaysia, Serdang, Selangor, Malaysia; ^3^ Department of Veterinary Physiology and Biochemistry, Faculty of Veterinary Medicine, Usman Danfodiyo University, Sokoto, Nigeria; ^4^ Department of Pharmacology and Toxicology, Faculty of Veterinary Medicine, Wroclaw University of Environmental and Life Sciences, Wroclaw, Poland; ^5^ Department of Biochemistry, Faculty of Chemical and Life Sciences, Usman Danfodiyo University, Sokoto, Nigeria; ^6^ Department of Surgery, Faculty of Clinical Sciences, Usman Danfodiyo University Teaching Hospital, Sokoto, Nigeria; ^7^ Department of Human Anatomy, Faculty of Basic Clinical Sciences, University of Maiduguri, Maiduguri, Nigeria; ^8^ Department of Veterinary Pathology, Faculty of Veterinary Medicine, University of Maiduguri, Maiduguri, Nigeria

**Keywords:** neuroprotection, dimethylsulfoxide, traumatic brain injury, oxidative stress, antioxidant ensymes, cognitive function, stress biomarker, anxiety

## Abstract

Traumatic brain injury (TBI) has been the result of neurological deficit and oxidative stress. This study evaluated the antioxidative neuroprotective property and learning and memory-enhancing effects of dimethyl sulfoxide (DMSO) in a rat model after the induction of TBI. 21 albino rats with 7 rats per group were used in this study. Group I was induced with TBI and treated with DMSO at 67.5 mg/kg orally once daily which started 30 min after the induction of TBI and lasted 21 days. Group II was induced with TBI but not treated while Group III was neither induced with TBI nor treated. Assessment of behavioral function (Learning and memory, anxiety and motor function), the level of an antioxidant enzymes and their gene expression (superoxide dismutase, catalase, glutathione peroxidase), the biomarkers of oxidative stress (malondialdehyde) and S100B levels as well as brain tissues histological studies were conducted. Administration of DMSO to rats with induced TBI has improved learning and memory, locomotor function and decreased anxiety in Group I compared to Group II. Moreover, the level of S100B was significantly (*p* < 0.05) lower in Group I compared to Group II. Treatment with DMSO also decreased lipid peroxidation significantly (*p* < 0.05) compared to Group II. There exists a significant (*p* < 0.05) increase in CAT, SOD, and GPX activities in Group I compared to Group II. Therefore, DMSO has demonstrated a potential antioxidative neuroprotective effect through its ability to increase the level of antioxidant enzymes which they quench and inhibit the formation of ROS, thereby improving cognitive functions.

## Introduction

Traumatic brain injury **(**TBI**)** is the physical and biochemical injury to the brain tissue, that occurs when an external force is applied to the head, resulting in various neurological dysfunctions (Maas et al., 2008). TBI is one of the leading causes of death and disability worldwide (Thurman, 2014). Annually, approximately 10 million individuals are affected by TBI worldwide, posing a significant health and socioeconomic challenge (Maas et al., 2017). In the United States alone, more than 2.8 million cases of TBI occur annually, and about 5.3 million Americans live with a long-term or life-long disability due to TBI (CDC, 2015). The most common causes of TBI in civilian populations are car accidents, violence, and falls (Faul et al., 2010). For those engaged in contact sports, mild TBI in the form of a concussion is common and sometimes accompanied by loss of consciousness (Zaza et al., 2022). However, bomb blasts and other forms of blasts have become the leading cause of TBI in the military and civilian population in a country affected by war (Levin and Robertson, 2012). In the African continent, road traffic accidents (RTA) are responsible for the occurrence of most head and spinal cord injuries (Thanni and Kehinde, 2006). In Nigeria, it was observed that RTA is the leading cause of the head trauma-related accident which may sometimes lead to death (Onyemaechi and Ofoma, 2016). TBI is characterized by the direct primary impact on the brain resulting from mechanical forces applied to the head at the time of trauma as well as delayed secondary damage induce by different cascade mechanisms of pathological processes that evolve later in the process (Xiong et al., 2013). Moreover, the secondary injury may arise following body reaction to resolve the primary insult impacted on the brain, and it includes complex biochemical events involving reduced cerebral blood flow, hypoxia, mitochondrial dysfunction/damage, intracellular calcium overload glutamate excitotoxicity, and neuroinflammation that all lead to neuronal cells death (McAlister, 2011). One key pathway is the primary shearing forces that are applied to neurons at the time of the traumatic episode. This causes huge ionic movement across the neuronal membranes, widespread depolarization, and rapid release of neurotransmitters (Resulting in excitotoxicity) from affected cells (Zhu et al., 2018). Afterward, a multitude biochemical events generate large amounts of toxic molecules such as nitric oxide, prostaglandins, free radicals, and inflammatory cytokines, which lead to a breakdown of the blood–brain barrier and the development of edema. The associated increase in intracranial pressure (ICP) and reduced cerebral blood flow, may then cause local hypoxia and ischemia with subsequent neuronal cell death via necrosis and apoptosis (Sahel et al., 2019). These processes and even the primary impact were found to generate reactive oxygen species (ROS) and other free radicals from the damaged brain tissues (Xiong et al., 2004). Furthermore, an imbalance in the rate of production of ROS may lead to oxidative stress and consequent damage to DNA, proteins, and lipids of the neuronal cells (Hole et al., 2011). The general endogenous antioxidant system consists of enzymatic antioxidants like superoxide dismutase (SOD), catalase (CAT), and glutathione peroxidase (GPx) which quench ROS generated in the system to protect against oxidative stress (He et al., 2017). Depletion of the endogenous antioxidant system has been observed to be associated with oxidative stress following brain damage under various pathophysiological conditions in animal models or human patients (Shivakumar et al, 1992). Experimental studies have shown that enhancing this endogenous antioxidant defense mechanism may be neuroprotective during injury (Ozdemir et al., 2005). Generally, their protective mechanisms involve inhibiting the synthesis and release of ROS, scavenging and neutralizing ROS as well as boosting the antioxidant system of the body (Sanmartín-Suárez et al., 2011).

Non-enzymatic antioxidants are substances that can scavenge free radicals and inactivate them in the system after administration (Flieger et al., 2021). Dimethyl sulfoxide (DMSO) is one of the most typical organic solvents used experimentally to dissolve insoluble chemical substances for *in vivo* and *in vitro* reasons (Kelava et al., 2011). It is an amphiphilic compound that can cross the blood-brain barrier (Broadwell et al., 1982). However, DMSO by itself was documented to exhibit several biological functions which might affect the pharmacological activity of chemical substances when employed as a vehicle (Kelava et al., 2011). It was reported to have antioxidants-based radical scavenging properties (Sanmartín-Suárez et al., 2011), and CNS modulating effects following neurologic abnormalities (Penazzi et al., 2017). The main goal of TBI treatment is to arrest the progression of secondary brain injury by targeting the pathways that may lead to inflammation, oxidative stress, and neuronal death (Gandhi and Abramov, 2012). Any therapeutic intervention that aims to prevent spatial memory and learning deficits and other brain functional abnormalities are crucial in the management of TBI. Several studies have reported the potential neurotherapeutic effect of DMSO that could be used in the management of various neurological conditions (Di Giorgio et al., 2008; Kelava et al., 2011; Sanmartín-Suárez et al., 2011; Jacob and Torre, 2015). Despite this, to our knowledge, the antioxidant-based neuroprotective properties of DMSO have not been previously investigated in rats models of TBI. Thus, this study aims to evaluate the antioxidant-base neuroprotective property and learning and memory-enhancing effects of dimethyl sulfoxide in the rat model of TBI.

## Material and methods

### Animals

The experiment was carried out using 21 healthy male albino rats (Wistar strain), 4 weeks of age, and weighing 180 g–200 g. The rats were obtained from a local supplier (Bistari Ltd.,) in Serdang, Selangor Malaysia, and were housed in the animal research facility at the Faculty of Health Sciences, University Putra, Malaysia. They were maintained under the regular 12/12 h light/dark cycle, with a room temperature ranging between 24^o^C and 26^o^C and relative humidity between 45% and 70%. They were fed with Rats commercial pellet diet (Gold coin^®^ feed), and provided with clean water *ad-libitum* for 2 weeks during which they were allowed to acclimate to the environment before commencing the experiment.

### Drug/chemicals

Pharmaceutical grade DMSO (99.9%) was obtained from Cayman^®^ Chemicals Company, Ann Arbor, United States. Superoxide dismutase (SOD), Catalase (CAT), Glutathione peroxidase (GPx), and lipid hydroperoxide (LPO) assay kits were obtained from Cayman^®^ chemical company, Ann Arbor, United States S100B ELISA kit was obtained from Fine Biotech, China. Ketamine hydrochloride was obtained from Rotexmedica^®^, Trittau, Germany. RIPA Lysis buffer kit was bought from Invitrogen, United States. Primers were sourced from Apical Scientific, Selangor, Malaysia, cDNA synthesis kit, and qPCR kit were obtained from Bioline reagent limited, United Kingdom.

### Experimental design

The rats were randomly divided into 3 groups (*n* = 7), namely: Group I, II, and III. The treatment group (Group I) was induced with TBI by weight drop method and treated with DMSO (67.5 mg/kg orally) for 21 days. Group II was traumatized but not treated (TNT) while Group III was neither traumatized nor treated (NTNT). Treatment was started 30 min after TBI and lasted for 21 days. The experiment was approved by the institutional animal care and use committee of the University of Putra Malaysia on 20 October 2017 with reference number UPM/ACUC/AUP-RO75/2017 as per the code of ethics of the World Medical Association (Declaration of Helsinki). The number of rats per group was adopted from our previous studies on TBI. The animals were randomly allocated to the various groups immediately after scoring them using the neurological severity score of rats. The behavioral studies was conducted using 7 rats per group and after that their number was reduced to 5 rats per group in which their brain tissue was used for biochemical studies and the remaining 2 for histological studies.

### Experimental procedures

#### Induction of traumatic brain injury

The experiment was carried out based on the “weight drop method,” where an acceleration impact device developed by Marmarou (1994) was used to inflict head injury on all experimental animals except for Group III (the negative control group). The experimental rats were properly restrained and anesthetized using the dissociative anesthetic agent Ketamine at a dose rate of 80 mg/kg body weight. The skull was exposed by a midline incision and a stainless steel disc measuring 10 mm in diameter and 3 mm in depth was cemented centrally along the control suture between the lambda and the bregma with a polyacrylamide adhesive. The experimental animals were secured in the prone position on a 10 cm deep foam bed. Injuries were induced by dropping an 80 g brass weight from a distance of 1 M. The stainless steel disc was immediately removed from the skull and the animal was allowed to recover in the cage. Based on the age and the body weight of the rats a drop of 80 g weighed brass from 1 m height can induce moderate injury as we have experimented before in our previous work.

#### Treatment

Treatment was instituted 30 min after the induction of TBI and lasted for 21 days where DMSO was administered orally to Group I at 67.5 mg/kg once daily. No any sign of toxicity was observed in the DMSO treated rats.

#### Learning and memory functions evaluation (morris water maze test)

Learning and memory abilities were tested in the Morris water maze on days 15–20 after TBI using the method described by Morris (1984). For each trial, the rat was placed in a circular tank with a hidden submerged platform. Each rat was tested on 4 trials a day, with each trial beginning in a new quadrant of the tank. The order in which the rat was placed in the 4 quadrants was randomized each day for each rat. The rat was allowed to swim to the platform and was left on the platform for 20 s before being removed. If the platform was not found after 120 s, the rat was placed on the platform for 30 s before being removed. A computerized video tracking system (Logitech digital camera attached to a computer with ANY-maze software) was used to record latency (time in seconds to find the platform), cumulative distance, and the average swim speed. After the first learning and acquisition trial period, each animal was given a probe trial, during which the platform was removed and the animal was allowed 60 s to search the pool. The amount of time that each animal spent in each quadrant was recorded (quadrant search time).

#### Assessment of locomotor activity and anxiety with open field test

The open field apparatus was constructed with white plywood and measured 100 × 100 cm with 30 cm walls. One of the walls was clear Plexiglas, so rats could be seen in both apparatuses. Blue lines were drawn on the floor with a marker and were visible through the clear Plexiglas floor. The lines divided the floor into sixteen 18 × 18 cm square. A central square (33 cm in diameter) was drawn in the middle of the open field and activity was recorded by a computer-operated digital camera system using ANY-maze software. Total distance (locomotor activity) and center distance (the distance traveled in the center of the arena) were recorded. The center distance was divided by the total distance to obtain a center distance to total distance ratio. The center distance to total distance ratio can be used as an index of anxiety-related responses. The total distance was used as an index of locomotor activity.

#### Sample collection

The rats were anesthetized using ketamine and blood was collected by cardiac puncture. Serum was harvested and kept at −20°C until further used.

#### Brain extraction and homogenization

This procedure was carried out according to the standard procedure described by Kim et al. (2016). Briefly, the skin was opened at the midline of the head cutting from the roof of the skull to the mid-eye area using micro dissecting scissors. After folding back the skin flaps with the scissors, the skull was cut at the midline fissure, without cutting into the brain tissue. The raised skull cap was removed with the curved forceps, applying slight pressure. The brain was then released from the skull cavity by running a micro spatula underneath and along the length of the brain from the olfactory lobes to the beginning of the spinal cord. After gently transferring the brain to a 60 mm petri dish, the tissues were rinsed with a phosphate-buffered saline (PBS) to remove any red blood cells and clots. Then the brains were transferred to a second petri dish and cut into small pieces in ice chilled 10% PBS solution, slices were sonicated for 45 min in 100 cycles. The extract was separated from the tissue by centrifugation at 1,500 rpm for 5 min, the supernatant was collected and used for the assay.

#### Estimation of calcium-binding protein S100B

The assay was done using an ELISA kit from Fine Biotech based on the principle of sandwich ELISA.

#### Estimation of superoxide dismutase, glutathione peroxidase, and catalase

These were assayed from the brain homogenate using Cayman’s Assay Kit, following the manufacturer’s instructions.

#### Estimation of lipid peroxidation

Lipid peroxidation as evidenced by the formation of thiobarbituric acid reactive substances (TBARS) was measured from the brain homogenate using Cayman’s Assay Kit.

### Gene expression study

#### Reverse transcriptase-polymerase chain reaction

The primers for each gene with their product size were depicted in Table 1 while the PCR cyclical condition in Table 2.

**Table 1:** Showing primers for each gene with their product size.

Gene	Forward primer	Reverse primer	Product size bp
GPX	5′GGA​CAT​CAG​GAG​AAT​GGC​AAG-3′	3′TCG​ATG​TCG​ATG​GTG​CGA​AA-5′	323
CAT	5′-GGT​CTG​GGA​CTT​CTG​GAG​T-3′	3′GAT​GGG​TAA​TTG​CCA​CTG​G-5′	285
SOD	5′ACT​TCG​AGC​AGA​AGG​CAA​GC-3′	3′TGA​GGT​CCT​GCA​GTG​GTA​CA-5′	133
Beta Actin	5′- ACA​ACC​TTC​TTG​CAG​CTC​CT-3′	3′CCC​ATA​CCC​ACC​ATC​ACA​CC-5′	200

**TABLE 2:** PCR cycling condition.

Cycles	Temperature(°C)	Time(S)	Steps
1	95	120	Initial denaturation
40	95	5	Denaturation
40	60	20	Annealing
40	60	20	Extension

#### RNA extraction

The brain homogenate from all the experimental animals was used for RNA extraction, cDNA synthesis, and PCR analysis. Briefly, 250 μl of the brain homogenate was dispensed in 1.5 ml sterile Eppendorf tubes and 750 μl of Trizol LS was added in a 1:3 ratio. The mixture was resuspended by several up and down pi-petting and allowed to stand for 15 min at room temperature. Chloroform (200 μl) was added to the mixture, shaken vigorously for 15 s, and then allowed to stand at room temperature for 5 min before centrifugation at 12,000 x g for 15 min at 4°C. The upper clear aqueous phase containing RNA was gently removed from the two organic and DNA phases into a new labeled 1.5 ml tube and was used for RNA precipitation. 500 µl of 100% isopropanol was added to each tube and allowed to stand at room temperature for 10 min before centrifugation at 1,200 x g and the isopropanol was discarded while the RNA was washed with 1,000 μl of 75% alcohol and centrifuged for 5 min at 7,500 x g. The alcohol was discarded and the RNA pellet partially dried inside level 2 biosafety cabinets for 5 min–10 min at the end of which 35 μl of sterile RNase-free water was added to resuspend the RNA for determination of concentration, purity, and subsequent use for downstream application. The RNA purity and concentration were determined using a NanoDrop^TM^ND-1000 UV-VisSpectrophotometer by ThermoFisher Scientific (Chomczynski and Mackey, 1995; Lawal et al., 2017).

#### cDNA synthesis

The extracted RNA was used to synthesize cDNA using Tetro cDNA synthesis kit (Bioline Pty limited, Australia) with the following reagent mixtures and conditions: RNA template (5 μl), RNase-free water (7.0 μl), and random oligomers (1.0 μl). The mixture was briefly centrifuged and incubated at 65^o^C for 2 min and rapidly chilled on ice for 5 min after which 4.0 μl of RT buffer, 1.0 μl of dNTPs, 1.0 μl of RiboSafe RNase inhibitor, and 1.0 μl of reverse transcriptase were added to bring the total reaction volume to 20 μl. The mixture was gently mixed, briefly centrifuged, and incubated at 37°C for 60 min after which the temperature was raised to 85°C for 5 min (Lawal et al., 2017).

#### Polymerase chain reaction amplification

The amplification was done with a qPCR machine Realplex from Eppendorf. The synthesized cDNA was used as a template for PCR amplification using a PCR kit from PCR Biosystem with the following reagents volume and concentrations as recommended by the manufacturer. 2X qPCRBioSYGreen Blue mix (10 μl), 10 μM Forward primer (0.8 μl), 10 μM Reverse primer (0.8 μl), Template DNA (1.0 μl) and PCR grade deionized water (up to 20 𝜇l). The same procedure was adopted to amplify all the three genes of interest and Beta-actin as a reference gene using the following primers as shown in Table 1 (Mullis et al., 1986).

The mixture was briefly centrifuged and incubated in PCR cycling conditions as shown in Table 2.

#### Expression of the genes

The threshold cycle (C_t_) values were measured to detect the threshold of each of three genes of interest and the Beta-actin gene in all samples. Each sample was measured in triplicate and normalized to the reference Beta-actin gene expression. The C_t_ value of each well was determined and the average of the three wells of each sample was calculated. Delta C_t_ (∆C_t_) for the test gene of each sample was calculated using the equation:

∆C_t_ = C_ttest_ gene–C_t_ Beta actin.

Delta delta C_t_ (∆∆C_t_) was calculated using the following equation:

∆∆C_t_ = ∆C_t_ t_est_ average - ∆C_t_ control group.

The fold change in the test gene expression was finally calculated from the formula:

Fold change = 2^-{∆∆Ct }^ (Livak and Schmittgen, 2001)**.**

#### Histological examination

Samples of the brain tissue extracted from all the experimental animals were fixed in 10% buffered formalin for 48 h. The fixed tissues were dehydrated in graded concentrations of alcohol (70%, 80%, 90%, and 100%) using an automatic tissue processor. The tissues were cleared using Xylene embedded with molten paraffin wax, blocked, and labeled appropriately. Tissue sections 5 µm thick were made from the embedded tissues using a microtome knife attached to a microtome. The sectioned tissues were mounted on a grease-free, clean glass slide, dried at room temperature, and stained with hematoxylin and eosin (H and E), Cresyl violet stain, and Belchowsky stain.

#### Statistical analysis

Results were analyzed using the statistical package—SPSS version 22. Results were expressed as means ± SD. Data were analyzed by one-way analysis of variance (ANOVA). If the F values were significant, the Tukey post-hoc test was used to compare groups. Gene expression fold changes by RT PCR were considered significant at a two-fold cut-off (*p* < 0.05).

## Results

### Learning ability after traumatic brain injury

**FIGURE 1:**
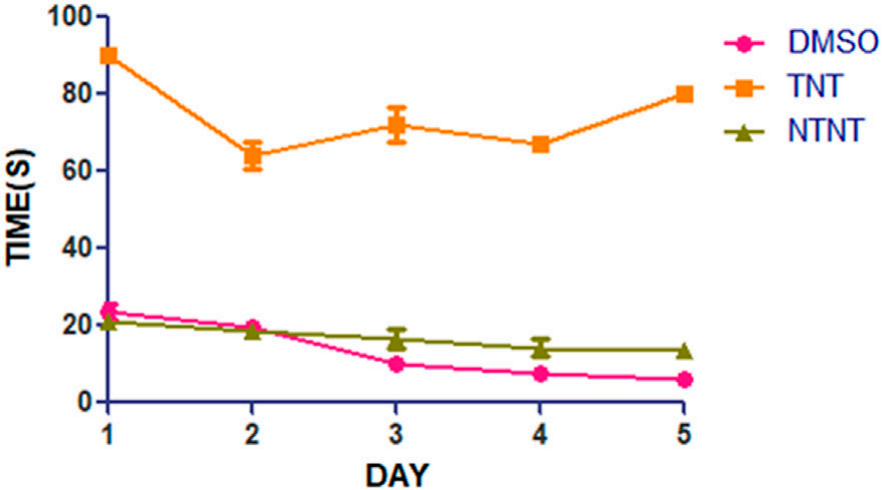
Effect of DMSO on learning skills in TBI induced rats; DMSO- dimethylsulfoxide, TNT- Traumatized Non-Treated, NTNT–Non traumatized non-treated. Data are expressed as the mean ± S.D. *n* = 7 per group. Data analyses were conducted using a one-way analysis of variance (ANOVA). Post hoc analyses were performed using Tukey’s multiple comparison tests when appropriate. *p < 0.05 compared to the traumatized non-treated and non-traumatized non treated groups.

From Figure 1, the traumatized non treated rat (group III) have escape latency that is not significant compared to DMSO treated group (Group I). By comparing the learning ability of rats in group I with those in traumatized non treated group (Group II), the rats in Group II have exhibited an increased (*p* < 0.05) escape latency than those in Group I.

### Memory function after traumatic brain injury

**FIGURE 2:**
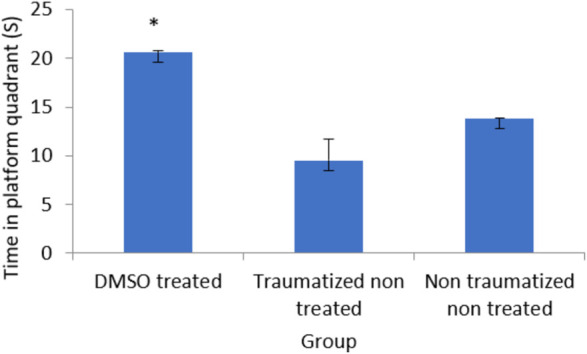
Effect of DMSO on memory in TBI induced rats; DMSO- dimethylsulfoxide, TNT- Traumatized Non-Treated, NTNT–Non traumatized Non treated. Data are expressed as the mean ± S.D. *n* = 7 per group. Data analyses were conducted using a one-way analysis of variance (ANOVA). Post hoc analyses were performed using Tukey’s multiple comparison tests when appropriate. *p < 0.05 compared to the traumatized non-treated and non-traumatized non treated groups.

**FIGURE 3:**
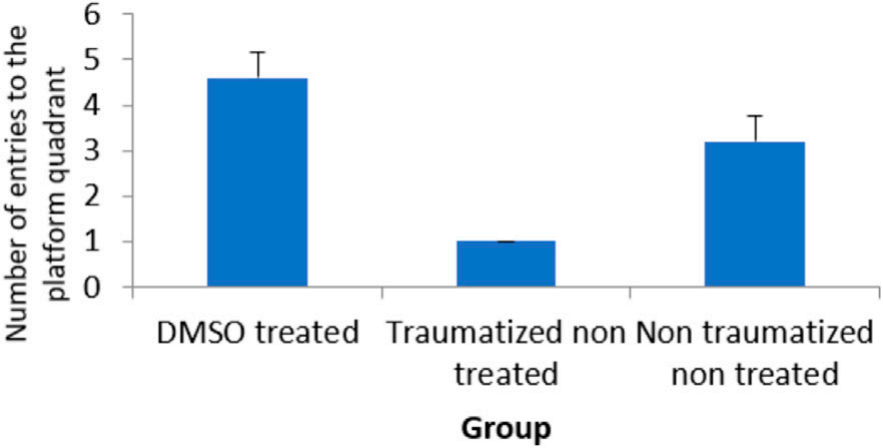
Effect of DMSO on locomotor activity in TBI induced rats; DMSO- dimethylsulfoxide, TNT- Traumatized Non-Treated, NTNT–Non traumatized Non treated. Data are expressed as the mean ± S.D. *n* = 7 per group. Data analyses were conducted using a one-way analysis of variance (ANOVA). Post hoc analyses were performed using Tukey’s multiple comparison tests when appropriate. *p < 0.05 compared to the traumatized non-treated and non-traumatized non treated groups.

From the result presented in Figure 2 on memory function after TBI, there is no significant (*p* > 0.05) difference between DMSO treated rats (Group I) and normal rats in non-traumatized non treated group (Group III) when the duration in the target quadrant and the number of entries are compared. However, Group I rats have demonstrated an increased duration (*p* < 0.05) in the target quadrant during the probe trial test when compared with traumatized non treated rats (Group II). This indicates that rats in Group I have remembered the escape route. Similarly, Figure 3 shows that there exists a significant difference (*p* < 0.05) in the number of entries into the target quadrant from where the escape route is located by rats in Group I as compared to rats in Group II.

### Locomotor activity after traumatic brain injury

**FIGURE 4:**
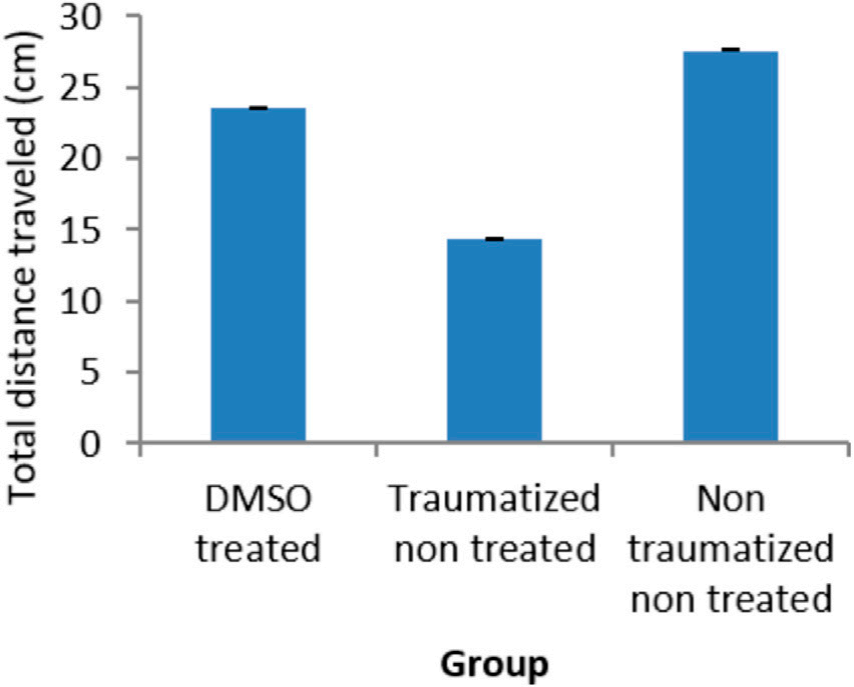
Effect of DMSO on locomotor activity in TBI induced rats; DMSO- dimethylsulfoxide, TNT- Traumatized Non-Treated, NTNT–Non traumatized Non treated. Data are expressed as the mean ± S.D. *n* = 7 per group. Data analyses were conducted using a one-way analysis of variance (ANOVA). Post hoc analyses were performed using Tukey’s multiple comparison tests when appropriate. *p < 0.05 compared to the traumatized non-treated and non-traumatized non treated groups.

Locomotor activity was evaluated with an open field test as presented in Figure 4 where non traumatized non treated rats in group III have demonstrated a normal locomotor function by having the longest distance traveled compared to traumatized non treated rats (Group II) and DMSO treated rats (Group I). While there is no significant difference (*p* > 0.05) between Group I and Group III, the difference between Group III and Group II is significant (*p* < 0.05). Group II rats also showed decrease in locomotor activity (Decreased distance traveled) compared to DMSO treated rats in Group I.

### Anxiety after traumatic brain injury

**FIGURE 5:**
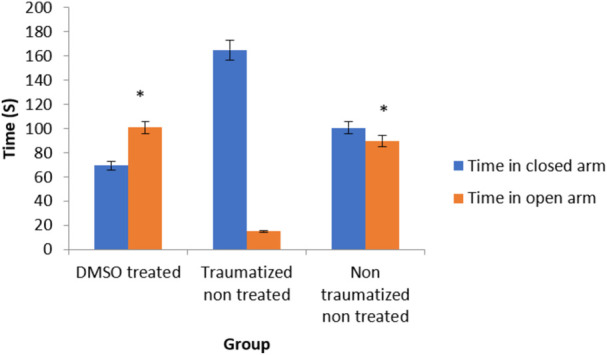
Effects of DMSO on anxiety-like behavior of rats induced with TBI; DMSO- dimethylsulfoxide, TNT- Traumatized Non-Treated, NTNT–Non traumatized Non treated. Data are expressed as the mean ± S.D. *n* = 7 per group. Data analyses were conducted using a one-way analysis of variance (ANOVA). Post hoc analyses were performed using Tukey’s multiple comparison tests when appropriate. *p < 0.05 compared to the traumatized non-treated and non-traumatized non treated groups.

The behavior of the experimental rats in the elevated plus maze was used to assess the anxiolytic effect of DMSO after TBI. The exploration of the open and closed arms was the measure of anxiety. There is no significant (*p* > 0.05) difference in the exploration of the open arm between the non-traumatized non treated rats (Group III) and DMSO treated rats (Group I) (Figure 5). Traumatized non treated rats (Group II) have a significantly (*p* < 0.05) decreased exploration of the open arm compared to the closed arm. At the same time, they have significantly (*p* < 0.05) increased exploration of the closed arm compared to DMSO treated rats (Group I) and non-traumatized non treated rats (Group III).

### Estimation of traumatic brain injury biomarker (S100 B)

**FIGURE 6:**
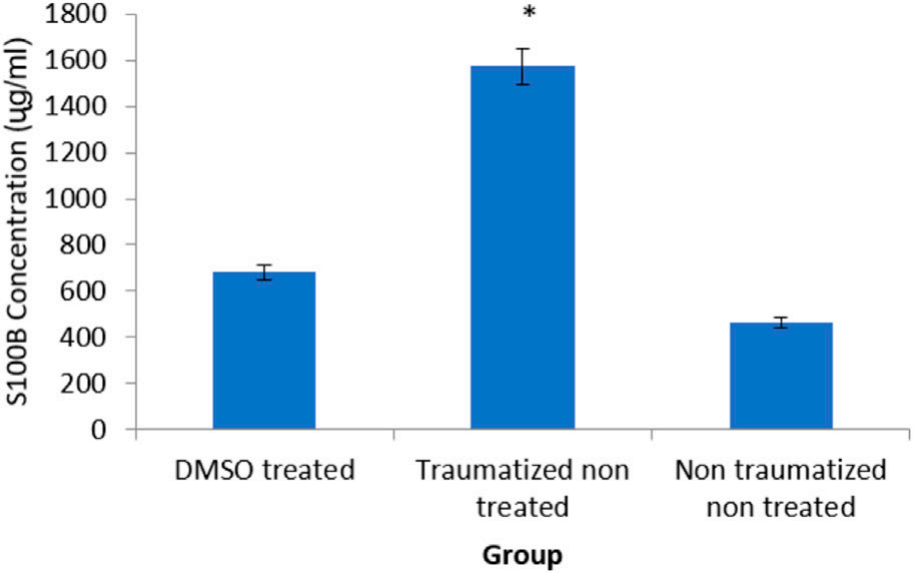
Effects of DMSO on the brain cortex concentration of S100 B in TBI induced rats. DMSO- dimethylsulfoxide, TNT- Traumatized Non-Treated, NTNT–Non traumatized non-treated. Data are expressed as the mean ± S.D. *n* = 5 per group. Data analyses were conducted using a one-way analysis of variance (ANOVA). Post hoc analyses were performed using Tukey’s multiple comparison tests when appropriate. *p < 0.05 compared to the traumatized non-treated and non-traumatized non treated groups.

Estimation of TBI biomarker the calcium-binding protein (S100 B) in the brain cortex of the experimental rats as shown was presented in Figure 6 The rats in non-traumatized non treated group (Group III) have a significantly (*p* < 0.05) lower concentration of S100B compared to traumatized non treated rats (Group II). DMSO treated group (Group I) also have significantly (*p* < 0.05) lower concentration compared to Group II. However, there is no significant (*p* > 0.05) difference between Group III and Group I.

### The effect of dimethyl sulfoxide on the activity of superoxide dismutase

**FIGURE 7:**
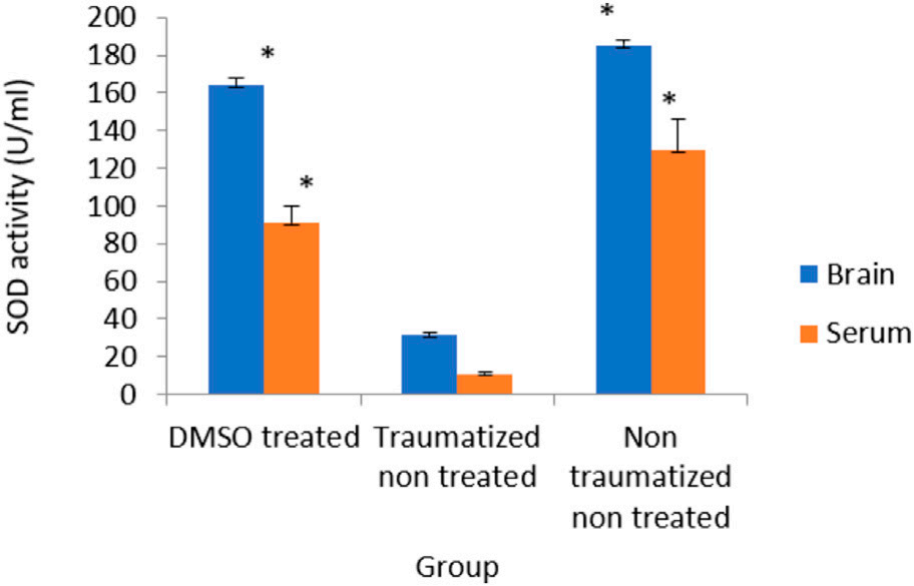
Effects of DMSO on the Activity of serum and brain cortex SOD in TBI induced rats; DMSO- dimethylsulfoxide, TNT- Traumatized Non-Treated, NTNT–Non traumatized Non treated. Data are expressed as the mean ± S.D. *n* = 5 per group. Data analyses were conducted using a one-way analysis of variance (ANOVA). Post hoc analyses were performed using Tukey’s multiple comparison tests when appropriate. *p < 0.05 compared to the traumatized non-treated and non-traumatized non treated groups.

Figure 7 shows the result of serum and brain cortex SOD activity. SOD activity observed in rats of non-traumatized non treated group (Group III) is significantly (*p* < 0.05) higher compared to traumatized non treated group (Group II) indicating that TBI caused a significant (*p* < 0.05) decrease in the activity of the enzyme in Group II. However, DMSO administration has significantly (*p* < 0.05) increased the activity of SOD in both serum and brain cortex of DMSO treated group (Group I) compared to Group II.

### The effect of dimethyl sulfoxide on the activity of glutathione peroxidase

**FIGURE 8:**
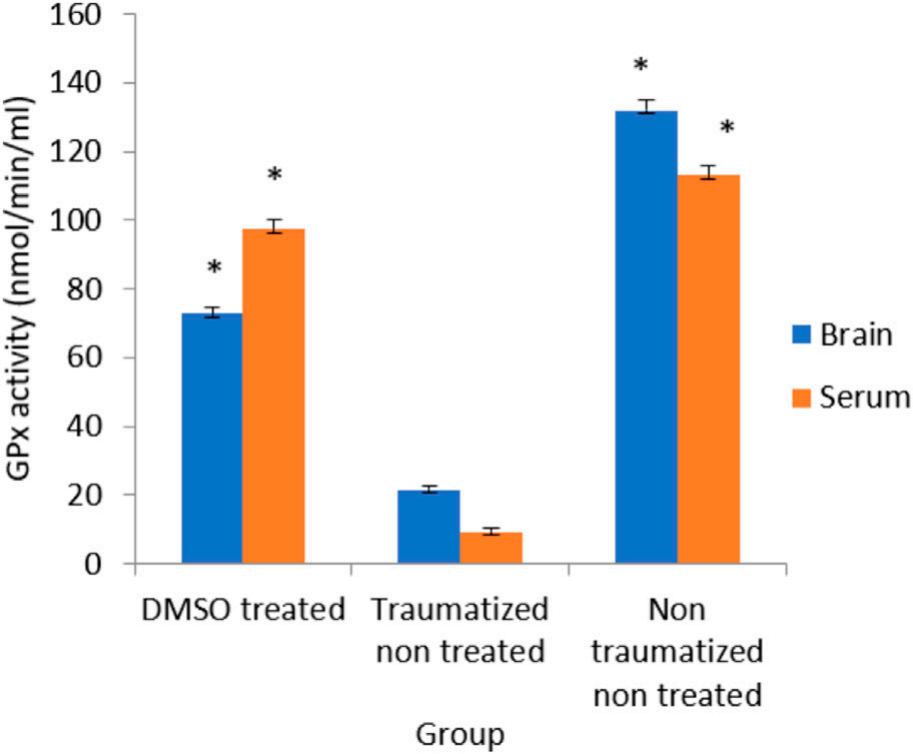
Effects of DMSO on the Activity of serum and brain cortex GPx in TBI induced rats; DMSO- dimethylsulfoxide, TNT- Traumatized Non-Treated, NTNT–non traumatized non treated. Data are expressed as the mean ± S.D. *n* = 5 per group. Data analyses were conducted using a one-way analysis of variance (ANOVA). Post hoc analyses were performed using Tukey’s multiple comparison tests when appropriate. *p < 0.05 compared to the traumatized non-treated and non-traumatized non-treated groups.

The effect of DMSO on the activity of GPx was presented in Figure 8 and the results showed that while the non-traumatized non treated rats have high enzyme activity, TBI caused a significant (*p* < 0.05) decrease in the activity of the enzyme in the traumatized non treated group (Group II). However, administration of DMSO to rats in Group I has significantly (*p* < 0.05) increased the enzyme’s activity in both blood and brain cortex. There is no significant difference in the serum level of GPx in group I compared to group III. However, in the brain cortex, the difference between Group I and non-traumatized non-treated rats (Group III) is significant (*p* < 0.05).

### The effect of dimethyl sulfoxide on the Activity of catalase

**FIGURE 9:**
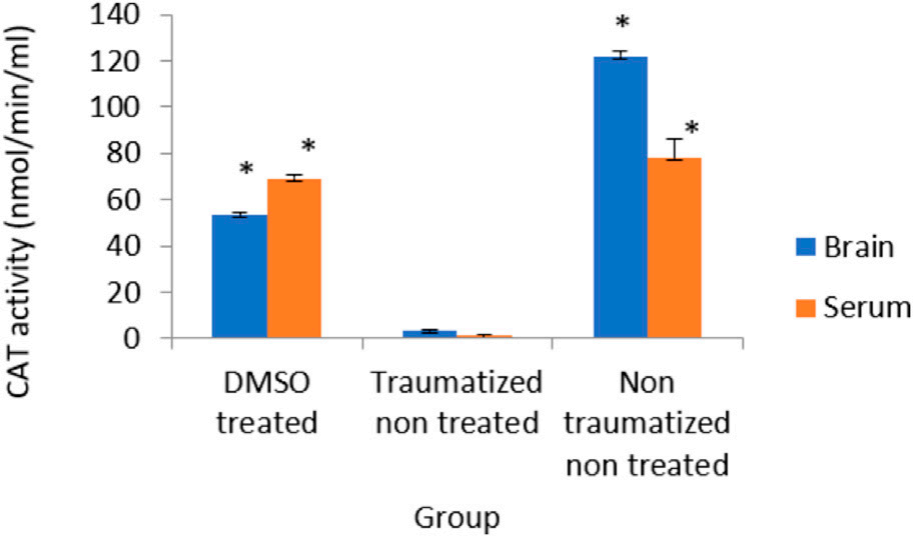
Effects of DMSO on the Activity of serum and brain cortex CAT in TBI induced rats; DMSO- dimethylsulfoxide, TNT- Traumatized Non-Treated, NTNT–Non traumatized non-treated. Data are expressed as the mean ± S.D. *n* = 5 per group. Data analyses were conducted using a one-way analysis of variance (ANOVA). Post hoc analyses were performed using Tukey’s multiple comparison tests when appropriate. *p < 0.05 compared to the traumatized non-treated and non-traumatized non treated groups.

Figure 9 shows the outcome of intervention with DMSO on the activity of CAT in TBI rats. The result from normal rats in the non-traumatized non-treated group (Group III) indicated high enzyme activity but the traumatized non treated group (Group II) shows a significantly (*p* < 0.05) decrease activity of the enzyme. The oral administration of DMSO increased the activity significantly (*p* < 0.05) compared to the traumatized non treated group (group II), and not significant compared to group III.

### The effect of dimethyl sulfoxide on brain tissue and serum levels of MDA

**FIGURE 10:**
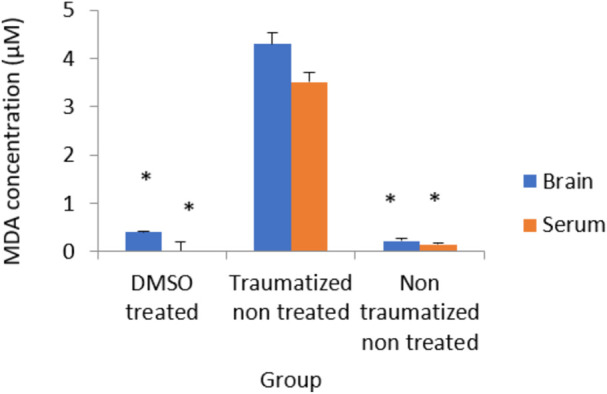
Effects of DMSO on the Activity of serum and brain cortex MDA in TBI induced rats; DMSO- dimethylsulfoxide, TNT- Traumatized Non-Treated, NTNT–Non traumatized non-treated. Data are expressed as the mean ± S.D. *n* = 5 per group. Data analyses were conducted using a one-way analysis of variance (ANOVA). Post hoc analyses were performed using Tukey’s multiple comparison tests when appropriate. *p < 0.05 compared to the traumatized non-treated and non-traumatized non-treated groups.

The results in Figure 10 indicated non traumatized non treated (group III) rats have low concentration of MDA while TBI caused a significant (*p* < 0.05) increase in the level of MDA in the brain cortex and serum of traumatized non treated (Group II) rats. The levels of MDA have decreased significantly (*p* < 0.05) in the samples obtained from rats in DMSO treated rats (Group I) but did not differ significantly from that of non-traumatized non-treated (group III) rats.

### Expression level of superoxide dismutase gene in traumatic brain injury rats treated with dimethyl sulfoxide

**FIGURE 11:**
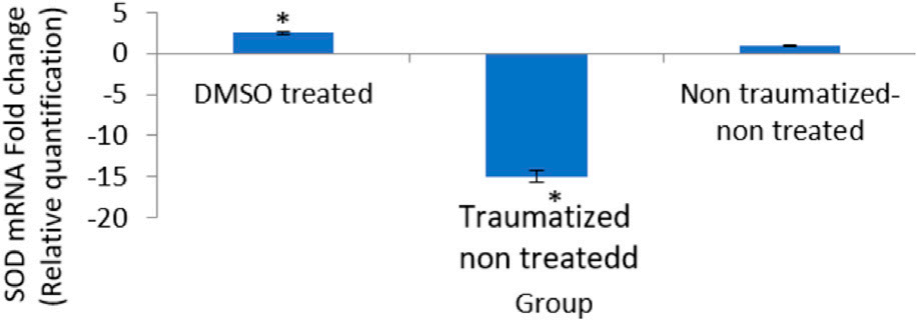
Effects of DMSO on the expression of SOD gene in brain cortex of TBI rats by relative quantification; DMSO- dimethylsulfoxide, TNT- Traumatized Non-Treated, NTNT–Non traumatized non-treated. Data are expressed as the mean ± S.D. *n* = 5 per group. Data analyses were conducted using a one-way analysis of variance (ANOVA). Post hoc analyses were performed using Tukey’s multiple comparison tests when appropriate. *p < 0.05 compared to the traumatized non-treated and non-traumatized non-treated groups.

Figure 11 is a result showing the expression levels of the SOD gene in all the experimental groups. The DMSO treated rats (Group I) showed 2 fold increase in the expression of the gene compared to traumatized non treated rats (Group II). While the gene expression level is shown to be down-regulated in the Group II rats.

### Expression level of glutathione peroxidase gene in the brain tissue of traumatic brain injury rats treated with dimethyl sulfoxide

**FIGURE 12:**
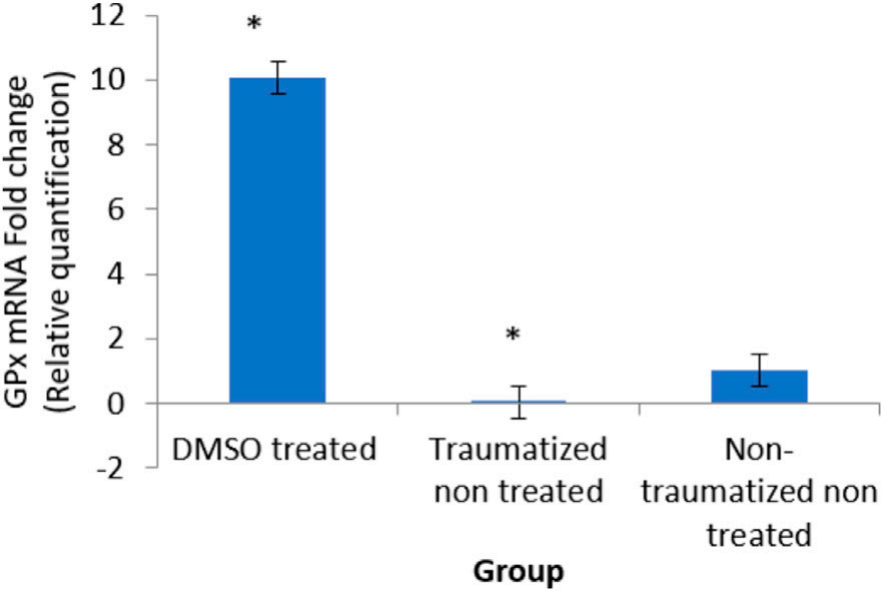
Effects of DMSO on the expression of GPx gene in brain cortex of TBI rats by relative quantification; DMSO- dimethylsulfoxide representing a treated group, TNT- Traumatized Non-Treated, NTNT–Non traumatized non-treated. Data are expressed as the mean ± S.D. *n* = 5 per group. Data analyses were conducted using a one-way analysis of variance (ANOVA). Post hoc analyses were performed using Tukey’s multiple comparison tests when appropriate. *p < 0.05 compared to the traumatized non-treated and non-traumatized non-treated groups.

As shown in Figure 12, comparing Group III with Group I and II, DMSO treatment in Group I caused an increase in the expression of the gene (10 fold). While TBI induction decreased the expression (0 fold) in rats in Group II.

### Expression level of catalase gene in traumatic brain injury rats treated with dimethyl sulfoxide

**FIGURE 13:**
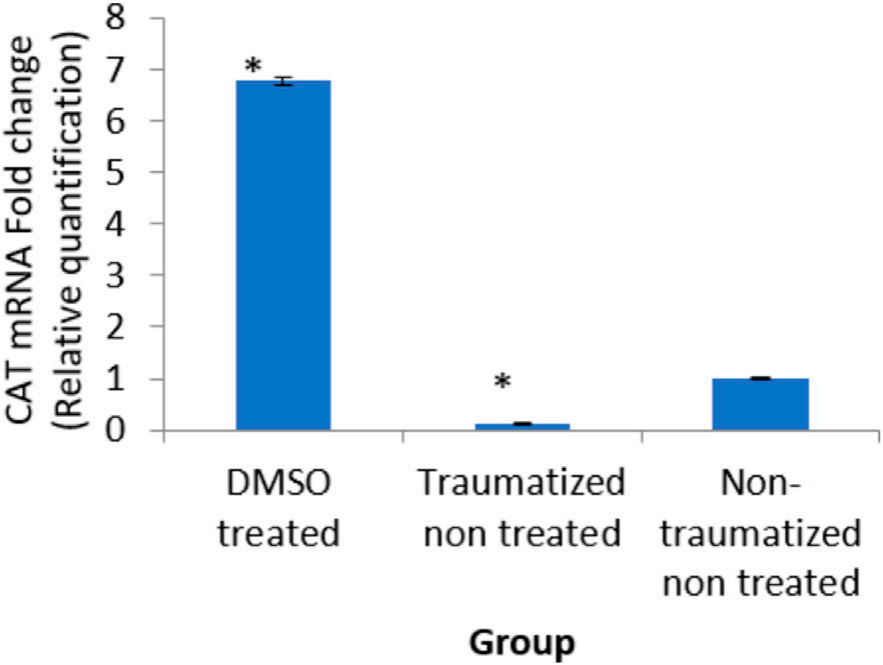
Effects of DMSO on the expression of CAT gene in brain cortex of TBI rats by relative quantification; DMSO- dimethylsulfoxide, TNT- Traumatized Non-Treated, NTNT–Non traumatized non-treated. Data are expressed as the mean ± S. D *n* = 5 per group. Data analyses were conducted using a one-way analysis of variance (ANOVA). Post hoc analyses were performed using Tukey’s multiple comparison tests when appropriate. *p < 0.05 compared to the traumatized non-treated and non-traumatized non-treated groups.

The result in Figure 13 indicated an up-regulation of the gene (6 fold) in the DMSO treated rats (group I) and decreased expression (0 fold) in traumatized non treated (group II) rats relative to the normal expression.

### Histology

**FIGURE 14:**
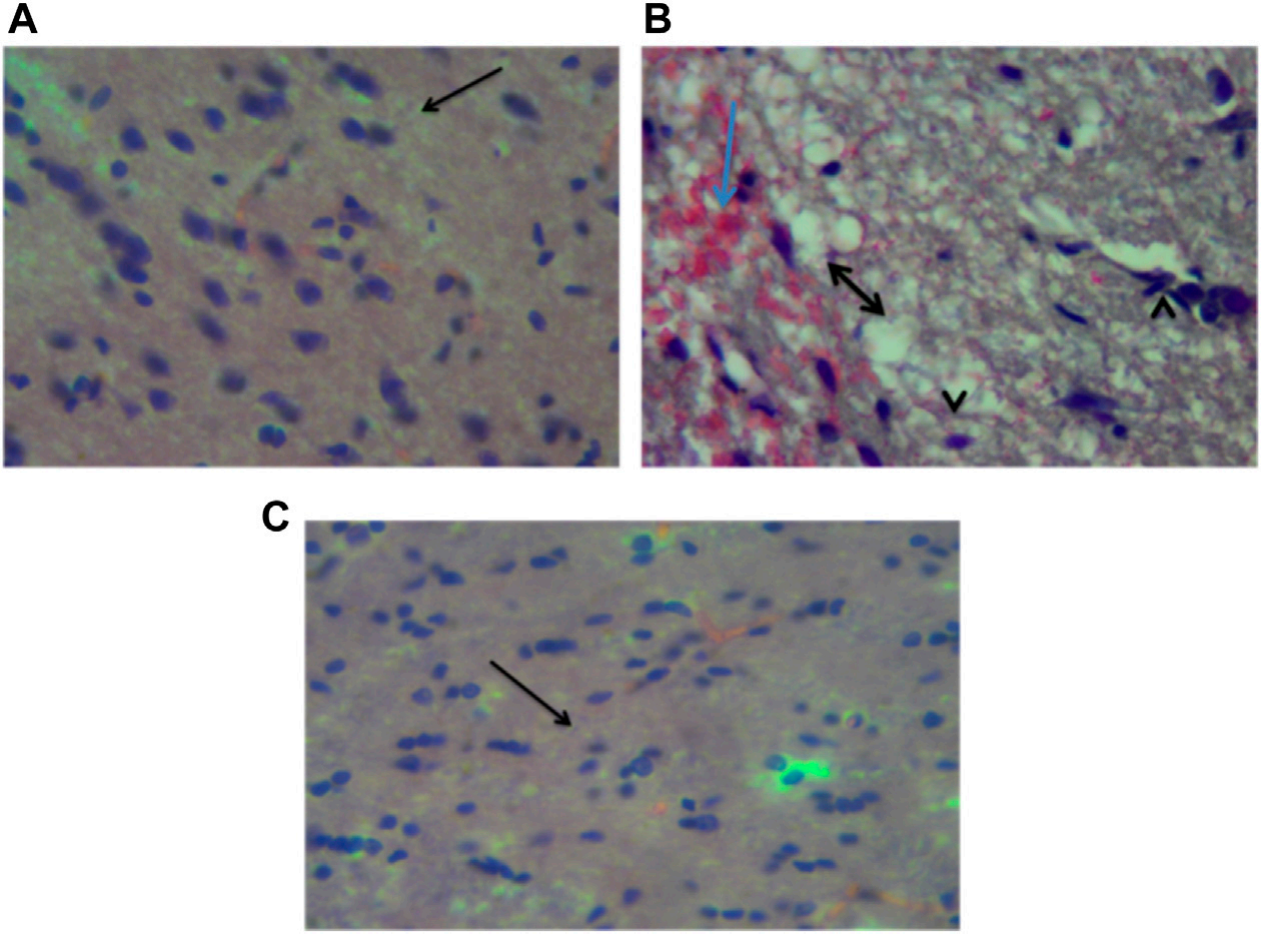
Photomicrograph of the brain (cerebrum) of **(A)** Non traumatized non treated (Group III) rats showing normal neurons of the cerebrum (arrow), **(B)** Traumatized non treated (Group II) showing spongiosis (double head arrow), hemorrhages (blue arrow) and necrosis of the neurons (arrowhead), **(C)** DMO treated rats (Group I) showing normalization of neuronal cells, H and E, X40 DMSO-Dimethylsulfoxide.

Figure 14 show the photomicrographs of brain tissues obtained from the three experimental groups that were stained with H and E stain. Figure 14 represents non traumatized non treated and examination of the slide revealed normal appearance or morphology of the neuronal cells, and layers, indicating the absence of injury or abnormality. Figure 14 represents traumatized non treated and examination of the slides shows the presence of inflammation, necrosis, congestion, and hemorrhage in the brain tissues. However, these lesions were not observed in Figure 14 which represents Group I that received DMSO treatment.

**FIGURE 15:**
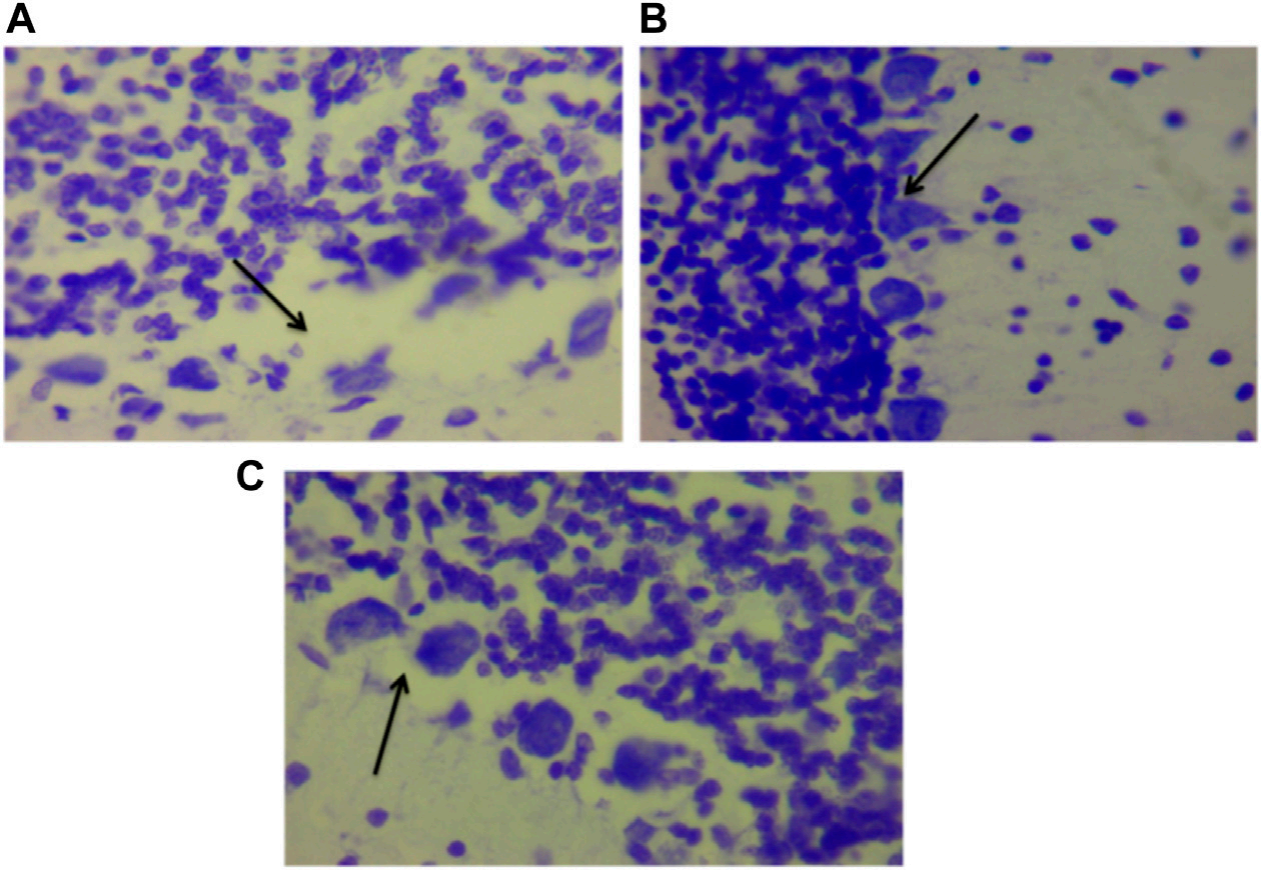
Photomicrograph of the cerebellum of **(A)** Traumatized non-treated (Group II) group showing loss of Purkinje cells (arrows), **(B)** Non traumatized non treated (Group III) rats showing a normal architecture of the cerebellum, and **(C)** DMSO treated rats (Group I) showing restored cell layer, DMSO dimethylsulfoxide, Nissl stain, X40.

Figure 15 show the photomicrograph of the cerebellum of rats for the three groups and were stained with cresyl violet (Nissl stain). In Figure 15 of group III (non-traumatized non-treated), the cell layers were observed to be normal. However, in Figure 15, the Purkinje cells layer of the cerebellum is shown to be distorted and cell loss was observed in the cerebellar tissues obtained from rats in Group II (traumatized non treated). Figure 15 (DMSO treated group) shows normalization of the cells layer with undistorted cells layer.

**FIGURE 16:**
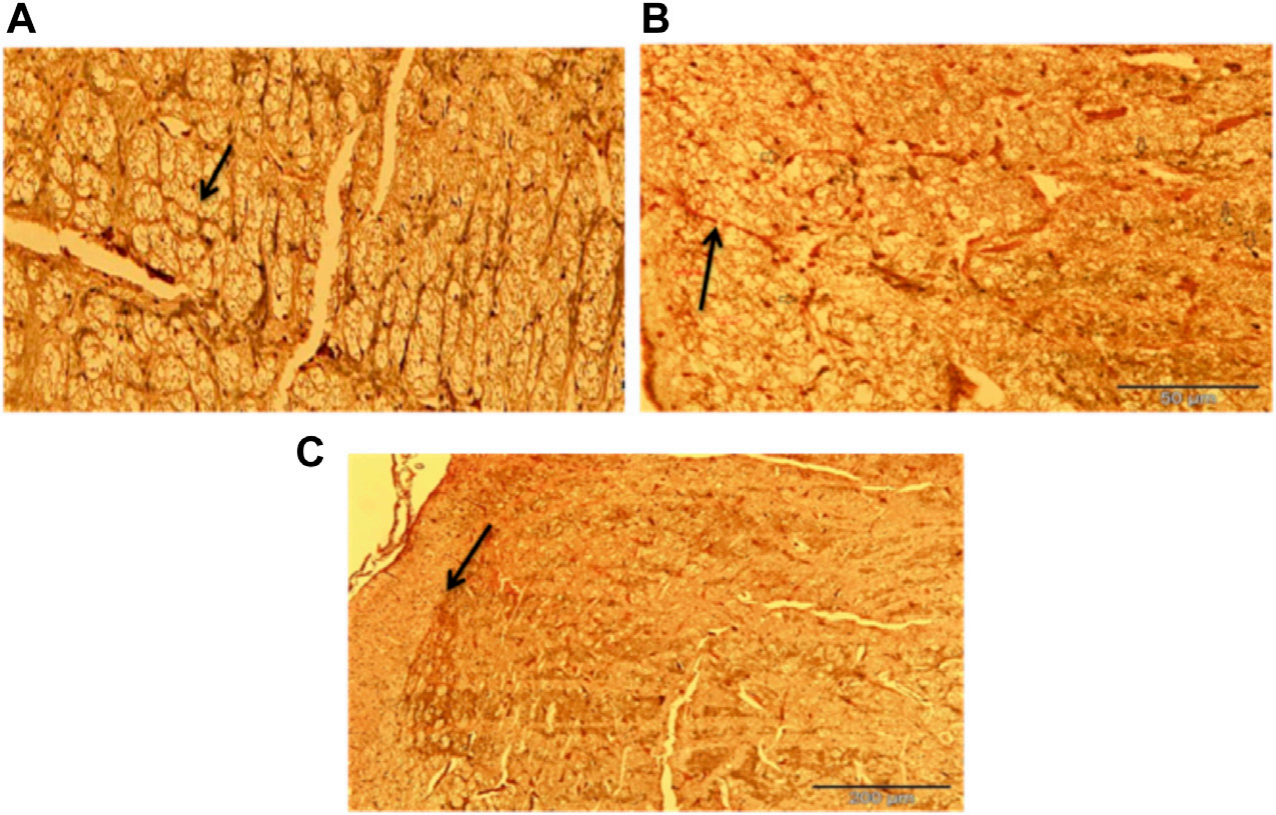
Photomicrograph of the cerebrum of **(A)** Non traumatized non treated (Group III) showing normal axon (arrow), **(B)**Traumatized non treated rats (Group II) showing axonal bulb (Thin arrow) and axonal break (Thick arrow), **(C)** DMSO treated rats (Group I) group showing normalization of the axon (arrow) Belchowsky stain ×40 DMSO dimethylsulfoxide.

Figure 16 represent the photomicrograph from the silver nitrate stained (Bielchowsky stain), and examination of the histological slides (Figure 16) that were obtained from rats in Group I (DMSO treated group) appears to be normal whereas slide (Figure 16) that was obtained from rats in Group II (traumatized non treated) shows the presence of axonal swelling (bulb) and axonal breaks. In Figure 16 which represents non traumatized non treated rats, the cortex shows normal axons.

## Discussion

TBI has been known to cause damage to the neurons, axons, dendrites, and microvasculature resulting in neurological dysfunction. Oxidative stress-mediated by reactive oxygen species (ROS) has been reported to be the key factor in the pathology of TBI (Cardenas-Rodriguez et al., 2013; Sahel et al., 2019). Several findings (Halliwell and Gutteridge, 2007; Waldbaum and Patel, 2010; Cardenas-Rodriguez et al., 2013; Cobley et al., 2018) substantiated the susceptibility of the brain to oxidative stress (Dasuni et al., 2013).

The findings of this study suggest that TBI by impact acceleration method in the rat induces a pronounced memory and learning deficit. The effect of DMSO on memory and learning skills in TBI was evaluated using the MWM test (Figures 1, 2). TBI was found to impair the rats’ ability to learn and retain the memory of the escape route in a morris water tank as observed in the rats in the traumatized non-treated rats (Group II). Interestingly, immediate treatment of TBI with DMSO (Group I) was found to attenuate the resultant brain tissue damage that may have led to memory decline. This shows that DMSO has an improved cognitive effect on the memory task by restoring memory function and increasing learning ability in the treated rats in Group I in comparison with Group II. Therapeutic strategies to prevent the spatial memory and learning deficits and other functional abnormalities observed in rodents following experimental TBI have focused on intervening in the secondary injury cascade mechanisms to prevent the neuronal loss, and axonal injury and improve synaptic plasticity (Mogensen et al., 2004; Galgano et al., 2017)**.** TBI induces abnormalities in neurotransmitter systems which also have vital roles in memory function (Mogensen et al., 2004; Galgano et al., 2017). One of the possible mechanisms of the effects of DMSO on learning and memory could be the role it plays in preventing mitochondrial damage during intracellular calcium overload and other destructive processes as well as preserving its function (Jacob and Torre, 2015; Davis and Raghu, 2022). Furthermore, oxidative stress is considered to be a probable cause of memory deficit due to impairment in hippocampal function which has an important role in learning and memory processes (Rekart *et al.,* 2007; Bulama *et al.,* 2017; Di Pietro et al., 2020; Davis and Raghu, 2022). Studies have reported that DMSO can have the antioxidative capacity to scavenge ROS and prevent lipid peroxidation (Di Giorgio et al., 2008; Jacob and Torre, 2015). Therefore, in the present study, oral administration of DMSO probably has mediated its function through the inhibition of oxidation by ROS thereby possibly preserving hippocampal function for good cognitive function. Moreover, DMSO has shown improvement in memory after observed memory deficit in rats with head injury (Jacob and Torre 2015). This effect could also be due to the modulatory role on neurotransmitter systems such as cholinergic and serotonergic systems because DMSO restores acetylcholinesterase activity, which has an essential role in learning and memory processes (Babri et al., 2015).

The result obtained for the open field test shows improvement in locomotor activity following treatment of TBI with DMSO (Figure 4). This may likely be due to the ability of DMSO to prevent the motor control areas in the brain from free radical damage and loss of locomotor function. Since dopamine has been associated with locomotor deficits, it can be speculated that its regulatory role on dopamine might have contributed to the improvement in locomotor activity.

The effect of DMSO on anxiety was tested by measuring the time spent in exploring the open arm versus the closed arm in EPM (Figure 5). The Traumatized non treated rats (Group II) exhibited a decreased exploration of the open arm. This anxiety-like behavior was abolished by DMSO where the rats treated with DMSO (Group I) showed indistinguishable behavior from the normal rats in the non-traumatized non-treated group in terms of increased exploration of the open arm. This may likely be attributed to the anxiolytic effect of DMSO through suppression of oxidative stress which induces anxiety (Penazzia et al., 2017). The study has shown that there is an increase in the production of ROS during anxiety (Guney et al., 2014). DMSO has also been shown to decrease Ca^2+^ responses to glutamate, protecting neurons from activation and excitotoxic death (Jacob and Torre 2015).

The high level of S100B in Group II (TNT) rats was observed to decrease significantly in the DMSO-treated rats (Group I). This indicates that the injury has reduced and the release of the protein is inhibited. S100B has been known to be a reliable biomarker of TBI owing to its release by activated glial cells during injury (Baker et al., 2009). It has been correlated with injury severity in hospitalized human patients (Hendoui *et al.,* 2013). High levels of S100B can stimulate inflammatory injury (Reali et al., 2005; Zou et al., 2022). S100 B has cytokine-like activities and can interact with the receptor for the advanced glycation end product (RAGE) (Mori et al., 2008; Zou et al., 2022). Therefore, the decreased concentration of S100B observed in the DMSO-treated rats could be due to its antioxidative effect. DMSO also has an anti-inflammatory effect which can prevent astrocytic damage and activation due to inflammatory mediated damage by ROS (Huang et al., 2014).

Following TBI, oxidative stress in this study was evaluated by measuring the levels of SOD, CAT, GPx, and MDA as indicators of enzymatic antioxidant activity and lipid peroxidation respectively. Treatment with DMSO caused a significant (*p* < 0.05) increase in the levels of antioxidant enzymes (SOD, CAT, and GPx) and a decreased concentration of MDA in the serum and brain of TBI rats in Group I compared to the TNT rats in Group II (Figures 7–10). These observed effects of DMSO could also be due to a single or combination of its following biological enhancing properties; free radical scavenging activity, suppression of intracellular calcium influx, blockage of Na^+^ channel activation, reduction of intracranial pressure, tissue edema, and inflammation (Santos et al., 2003).

DMSO is a powerful free radical scavenger and its utilization in a condition of oxidative stress may prevent the accumulation of free radicals that consumed the enzymatic antioxidants and reduce their levels (Cos et al., 2017). Reduced levels of the antioxidant enzyme result in oxidative stress (Shivakumar et al., 1992; Ahmad Amar et al., 2019). The generation of free radicals by the cells within a biological system also causes lipid peroxidation resulting in increased release of MDA a by-product of lipid degradation. Therefore the scavenging effect of DMSO could have reduced the chain reaction of lipid peroxidation by free radicals. DMSO has been shown to suppress, in a reversible manner the excessive calcium influx into cells which stimulates excessive free radicals production and channel-opening of the ionotropic receptor channels N-methyl-D-aspartate (NMDA) and a-amino-3-hydroxy-5-methylisoxazole-4-propionate (AMPA), which are known both to be activated by glutamate during oxidative stress (Jacob and Torre 2015). This “excitotoxic” process by glutamate can damage neurons and promote lipid peroxidation. (Camici et al., 2006). reported that DMSO also can block Na^+^ channel activation The Na^+^channel is activated by the physical impact on the brain tissue in TBI which leads to initiation of action potential and loss of membrane permeability culminating in necrosis and free radical generation (Huang et al., 2014; Gerhalter et al., 2021). (Jacob and Torre, 2009) reported a reduction in intracranial pressure, tissue edema, and inflammatory reactions by DMSO following TBI and these could further support its neuroprotective effects. The neuroprotective effect of DMSO observed in this study agrees with other studies reported by (Torre et al., 1973) who first reported the potential therapeutic effect of DMSO in central nervous system injury; Nagel et al., (2007) and Walter et al. (2020) reported that DMSO has a neuroprotective effect in experimental cerebral injury following cerebral ischemia (Sulieman et al., 2015) also reported that DMSO protected neuronal cells from damage in experimental head injury. The up-regulation of the SOD, GPx, and CAT genes in the DMSO treated Group of rats (Figures 11–13), with a concomitant increase in serum and brain of the rats treated with DMSO (Figures 11–13), further confirmed that the increase in the enzymatic activities is a result of increased synthesis which is influenced by DMSO. This could explain the decreased lipid peroxidation and neuroprotection observed. Though the mechanism for this up-regulation is not clear it can be speculated that DMSO might have influenced this through the transcription factors that regulate antioxidant genes. (Liang 2011 and Wuputra et al. (2022) reported that DMSO was observed to increase the expression of Nrf2. Transcription factors such as nuclear factor erythroid related factor (Nrf2), nuclear factor kappa B (NF-kB), and AP-1 were reported to be activated by antioxidants such as curcumin, resveratrol, and quercetin (Wong et al., 2018). Nrf2 once activated, binds to the antioxidant response element (ARE) located at the regulatory regions of the genes of the antioxidant enzymes and induces transcription of the antioxidants genes (Morinho et al., 2014). The photomicrographs of the brain tissue slide from the rats in Group II indicated the occurrence of injury on the brain ranging from rupture and sloughing off of the meninges to congestion, hemorrhage, and inflammation. These lesions were observed in the cerebrum, cerebellum, and meninges indicating the induction caused diffuse brain injury (Figures 14–16). The observed lesions such as rapture, sloughing off of meninges, and hemorrhage was indicative of primary injury while the inflammatory responses that may cause neuronal loss, axonal injury, and impairment of synaptic plasticity indicate the secondary injury process (Andres et al., 2014). The absence of observed primary and secondary lesions in Group I that received DMSO treatment possibly is an indication of normalization of the brain tissue or reduction of the severity of the lesions probably due to some improvement as a result of the intervention.

**FIGURE 17:**
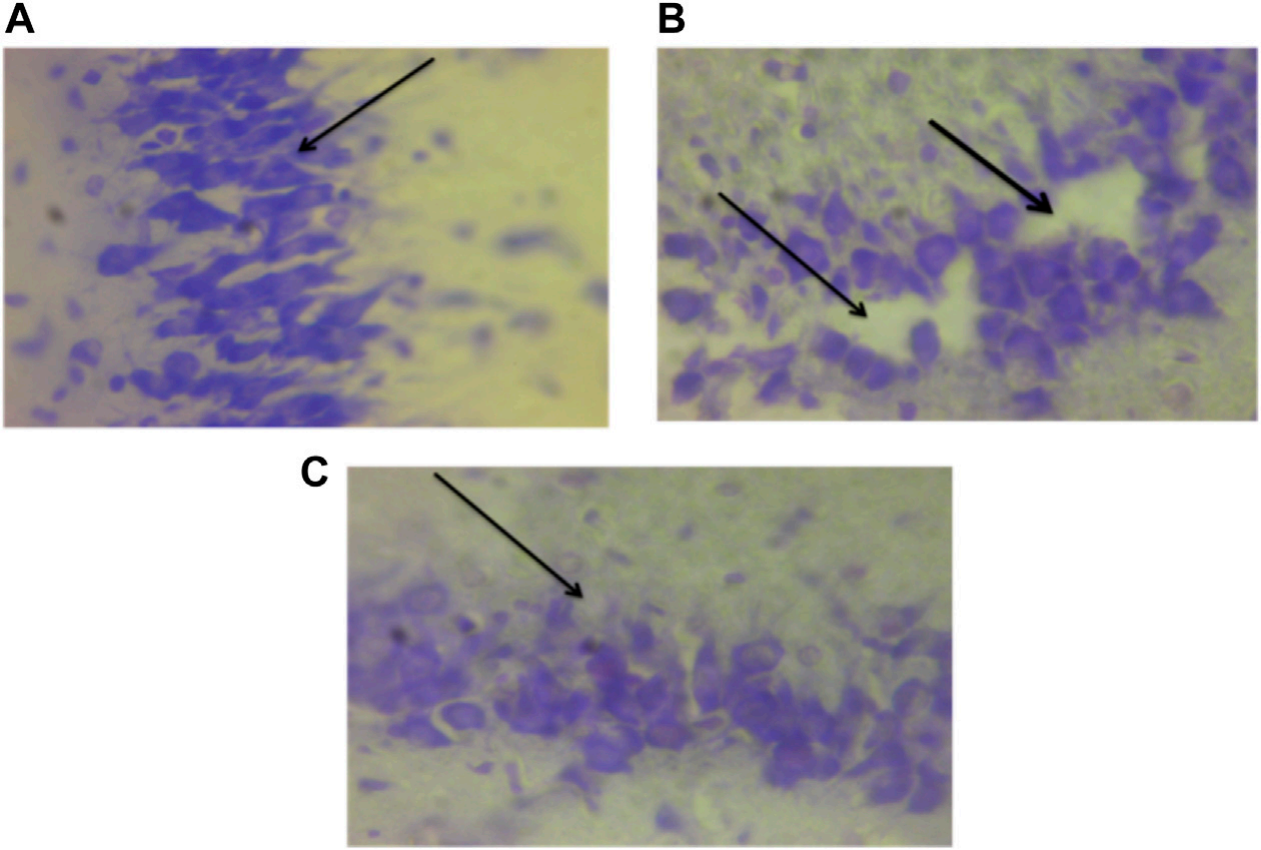
Photomicrograph of the hippocampus of TBI and normal rats; **(A)** Non traumatized non treated (Group III) with intact cell layers, **(B)**Traumatized non treated rats (Group II) showing distorted layers and cell loss, and **(C)** DMSO treated rats (Group I) sowing undistorted pyramidal cell layer. DMSO–dimethylsulfoxide. Nissl stain (×40).

The hippocampus is an essential brain region concerned with several vital functions, such as cognition, learning, and memory. In several animal models, such as hypoxia and ischemia, the conclusion is that the hippocampus is the most vulnerable brain region to oxidative damage and degeneration due to the over-activation of glutamate receptors, activation of caspases, and subsequent cell death (Shah et al., 2015). Therefore the hippocampal lesion observed in this study (Figure 17) might be responsible for the impairment in memory and learning exhibited by the rats in Group II and the protective effect of DMSO may perhaps be responsible for the enhancement of learning and memory observed in Group I. Axonal injury caused by TBI is the key feature of diffuse brain damage. Rapid acceleration/deceleration induces shearing forces on the axons which cause stretching and tearing damage. Compromised axonal protein transport post-TBI (Lifshitz et al., 2007) and intracellular protein accumulation such as Beta-amyloid precursor protein (β-APP) at the point of axonal breakage forms retraction bulbs/swelling (Risdall et al., 2011). Axonal injury is linked with impairment in memory, sensorimotor, and learning functions (Sidaros et al., 2007; Yang et al., 2022).

## Conclusion

From this study, it can be concluded that induction of TBI by the weight drop method causes neurological deficits and oxidative stress. However, treatment with DMSO was shown to improve functional recovery of cognitive function in rats after TBI through the enhancement of the body’s antioxidant defense system where it suppressed lipid peroxidation and reduces oxidative stress, and neuropathology. These neuroprotective effects can be a result of the enhanced antioxidant properties of DMSO which is mechanistically associated with the up-regulation of the SOD, GPx, and CAT genes. These promising results could suggest that antioxidants supplementation can be useful in patients with TBI as part of the therapeutic strategy for targeting secondary injury of TBI.

## Data Availability

The raw data supporting the conclusions of this article will be made available by the authors, without undue reservation.

## Abbreviations

TBI, traumatic brain injury; ROS, reactive oxygen species; SOD, superoxide dismutase; CAT, catalase; GPX, glutathione peroxidase; S100B, S100 calcium-binding protein B; RTA, road traffic accidents; ARE, antioxidant response element; MWM test, morris water maze test.
